# Familiarity with Population Health Management Terminology and Attitudes Toward PHM-Aligned Principles Among Primary Care Attendees in Riyadh, Saudi Arabia: A Cross-Sectional Study

**DOI:** 10.3390/healthcare14121608

**Published:** 2026-06-08

**Authors:** Nurah Alamro, Abdulaziz Alderaywsh, Abeer Awwad, Leena R. Baghdadi

**Affiliations:** 1 Department of Family and Community Medicine, College of Medicine, King Saud University, Riyadh 11461, Saudi Arabia; nmalamro@ksu.edu.sa (N.A.); lbaghdadi@ksu.edu.sa (L.R.B.); 2 Department of Family and Community Medicine, King Saud University Medical City, King Saud University, Riyadh 11461, Saudi Arabia; 3 College of Medicine, King Saud University, Riyadh 11149, Saudi Arabia; abawwad.md@gmail.com

**Keywords:** population health management, primary care, health literacy, patient activation, preventive services, Saudi Arabia

## Abstract

**Highlights:**

**What are the main findings?**

- Most participants were unfamiliar with the term population health management, and only a minority reported participation in preventive health programs.
- Agreement with PHM-aligned principles assessed by the study-developed questionnaire was generally favorable and was positively associated with health literacy and patient activation.

**What are the implications of the main findings?**

- These findings indicate that familiarity with PHM terminology remains limited in this primary care sample.
- The findings highlight limited familiarity with PHM terminology and support the need for further research using more rigorously validated PHM-specific measures.

**Abstract:**

**Background/Objectives**: Population health management (PHM) emphasizes prevention, coordinated care, and population-level approaches to improving health outcomes. However, patient familiarity with PHM terminology and attitudes toward PHM-aligned principles remain underexplored in primary care settings. This study assessed familiarity with the term PHM, attitudes toward PHM-aligned principles, and participation in preventive health programs among adult primary care attendees in Riyadh, Saudi Arabia, and examined their associations with health literacy, patient activation, and sociodemographic characteristics. **Methods**: A cross-sectional survey was conducted among adult attendees of primary care outpatient clinics at King Saud University Medical City, Riyadh, Saudi Arabia, between 1 October and 1 December 2025. Participants completed a self-administered Arabic questionnaire that included PHM awareness items, Questionnaire Assessing Population Health Management-Aligned Principles (PHM-Q), a study-developed 7-item instrument assessing agreement with PHM-aligned principles, the HLS-Q12, and the PAM-13. Descriptive statistics, chi-square tests, Mann–Whitney U tests, Kruskal–Wallis tests, Spearman correlation analyses, and internal consistency assessment were used. **Results**: A total of 286 participants were included. Familiarity with the term PHM was limited, with 24.8% reporting prior exposure. Participation in preventive health programs was also limited, as only 17.5% of participants reported having participated. In contrast, agreement with PHM-aligned principles was generally favorable (PHM-Q mean 3.86, SD 0.57). PHM-Q scores correlated positively with health literacy (ρ = 0.548, *p* < 0.001) and patient activation (ρ = 0.433, *p* < 0.001). Participation in preventive health programs was significantly associated with higher health literacy and higher patient activation. **Conclusions**: These findings should be interpreted as reflecting favorable attitudes toward PHM-aligned principles within this sample rather than formal PHM literacy, operational understanding, or readiness for PHM implementation. Because the study-developed questionnaire was exploratory and not psychometrically validated beyond internal consistency, the results should be understood as descriptive and hypothesis-generating.

## 1. Introduction

Healthcare systems worldwide face increasing pressures from rising costs, population aging, and the growing burden of chronic diseases. The number of individuals aged 65 years and older is projected to more than double from 703 million to 1.5 billion by 2050, and their proportion of the total world population is expected to increase from 6% to 16%. Concurrently, noncommunicable diseases caused 43 million deaths in 2021 and accounted for 75% of non-pandemic-related deaths worldwide [1,2]. In Saudi Arabia, noncommunicable diseases (NCDs) account for 73.2% of all deaths, and their overall burden is projected to more than double over the next three decades if no action is taken. The current health expenditure reached 5.69% of GDP in 2023, and the direct costs of NCDs are estimated at 23% of current health expenditure [3,4]. These trends have intensified interest in prevention-oriented care models that seek to improve population health while maintaining healthcare sustainability.

Population health is the health outcomes of a group of individuals, including the distribution of such outcomes within the group [5]. Population health management (PHM) has been defined inconsistently across the literature. Most definitions emphasize improving population health, whereas quality of care and cost containment are mentioned less consistently, showing that many definitions are not fully aligned with the Triple Aim [6]. PHM is a patient-centered, integrated care delivery model built on aligned incentives and coordinated, collaborative processes, guided by evidence-based prevention and disease management protocols [7].

There is growing recognition that improving health outcomes for heterogeneous populations such as individuals with chronic conditions requires integration across healthcare, public health, social care, and welfare systems [8,9]. PHM initiatives are designed to bridge these sectors and facilitate coordinated, continuous care [10,11].

Primary care is a particularly important setting for PHM because it enables proactive, people-centered, and targeted care for defined population groups, while supporting coordination and attention to broader health needs [12]. PHM can strengthen primary care by enabling providers to identify population groups with similar needs and deliver targeted, proactive, and coordinated services [13].

Health literacy is relevant to PHM because it influences patients’ ability to access, understand, appraise, and apply health information for healthcare decision-making, disease prevention, and health promotion [14]. Patient activation reflects the knowledge, skills, and confidence needed for individuals to manage their health and healthcare [15]. Higher patient activation has been associated with better health outcomes and care experiences, making it relevant to prevention-oriented and PHM-aligned care [16].

In Saudi Arabia, recent health-sector reforms have emphasized person-centered, preventive, and integrated services to address major public health challenges, including chronic diseases and preventable mortality [17,18]. Furthermore, a Saudi Public Health Authority–World Bank report indicates that noncommunicable diseases in Saudi Arabia impose substantial economic burdens on individuals, government budgets, productivity, and the broader economy, reinforcing the importance of prevention-focused reforms and a patient-centered integrated model of care [4]. The existing literature has examined PHM as a system-level implementation and service-design strategy, as well as through models of integrated care, with less attention to patient-facing perspectives in routine primary care settings [19,20]. Although PHM has been increasingly incorporated into health policy frameworks and system reform efforts, familiarity with PHM terminology and attitudes toward PHM-aligned principles among primary care patients remain underexplored. In this study, agreement with PHM-aligned principles was examined using a study-developed exploratory questionnaire based on PHM-aligned principles described in the literature, rather than formal PHM literacy, operational understanding, or readiness for PHM implementation. Therefore, this study aimed to assess familiarity with the term PHM, agreement with PHM-aligned principles, participation in preventive health programs, and their associations with health literacy, patient activation, and sociodemographic characteristics among primary care attendees in Riyadh, Saudi Arabia. The present study was therefore designed as a context-specific descriptive exploration rather than as an implementation-science evaluation of PHM.

## 2. Materials and Methods

### 2.1. Study Design and Setting

This study used an analytical cross-sectional design to assess familiarity with the term PHM, agreement with PHM-aligned principles, and participation in preventive health programs among adult primary care attendees. It was conducted in Family Medicine, Primary Care, and VIP outpatient clinics at King Saud University Medical City, and data were collected using a survey from 1 October 2025 to 1 December 2025. This study is reported in accordance with the STROBE guidelines for cross-sectional observational studies.

### 2.2. Participants and Sampling

Participants were adults (≥18 years) attending all primary care outpatient clinics at King Saud University Medical City during the study period. Eligible participants were required to be able to read Arabic, while individuals who were severely ill, required urgent care, or were unable to complete the survey or who had cognitive impairment preventing informed participation were excluded. Participants were recruited using a convenience sampling approach, in which eligible patients were enrolled consecutively from the clinic. Trained data collectors approached eligible attendees in the waiting area during the study period and invited them to participate. A total of 301 questionnaires were distributed: 1 individual declined to participate, and 14 questionnaires were excluded because of incomplete responses, resulting in an analytic sample of 286 participants.

### 2.3. Variables and Measurements

The main study measures included familiarity with the term PHM, agreement with PHM-aligned principles, and participation in preventive health programs. Agreement with PHM-aligned principles was assessed using the Questionnaire Assessing Population Health Management-Aligned Principles (PHM-Q), a study-developed 7-item instrument. The PHM-Q items covered key domains aligned with PHM principles, including prevention and early intervention, improvement in population health, care coordination, person-centered and holistic care, value-based care, and data-driven decision-making [5,20,21]. The instrument was intended as an exploratory attitudinal measure of agreement with PHM-aligned principles described in the literature, rather than as a validated measure of PHM literacy, PHM readiness, or understanding of PHM as a structured organizational or implementation model. Responses were recorded on a 5-point Likert scale, and total PHM-Q scores were calculated as the mean of the 7-item responses, with higher scores indicating greater agreement with PHM-aligned principles. No exploratory or confirmatory factor analysis was performed, and no external validation procedures were undertaken in the present study. Additional study measures included prior exposure to the term PHM and self-reported participation in preventive health programs. Prior exposure to the term PHM was assessed using the item ‘Have you heard about Population Health Management (PHM)?’ Participation in preventive health programs was assessed using the item ‘Have you participated in any preventive health program provided by the clinics (e.g., screening, vaccination, counseling)?’. Key predictors comprised demographic characteristics, health literacy, and patient activation. Health literacy was measured using the validated Health Literacy Survey Questionnaire (HLS-Q12) [22]. Item coding and score computation were performed so that higher mean scores reflected higher health literacy. Patient activation was assessed using a 13-item questionnaire based on the PAM-13 format [15], with response options ranging from strongly disagree (1) to strongly agree (4), as well as a not applicable option. For descriptive and association analyses, mean item scores were used, and questionnaires with more than three not applicable responses were excluded from analyses involving patient activation. The questionnaire items underwent expert review to assess face and content relevance, and a pilot study with 20 participants was conducted prior to the main data collection. Internal consistency reliability for the PHM-Q, HLS-Q12, and PAM-13 was assessed using Cronbach’s alpha. For the PHM-Q, internal consistency was assessed as a preliminary descriptive property only and was not interpreted as evidence of construct validity. All participants completed the same self-administered questionnaire under similar conditions. The PHM-Q item text is provided in Table A1.

### 2.4. Bias and Sample Size

Potential sources of bias included selection bias related to single-center clinic-based recruitment and self-report bias associated with questionnaire responses, including possible recall and social desirability effects. Efforts were made to minimize these biases by including eligible participants attending the clinic during the study period and by anonymous questionnaire administration. Sample size was calculated assuming a PHM awareness prevalence of 50%, a 95% confidence level (α = 0.05), and a 5% margin of error. This calculation was based on the primary descriptive objective of the study rather than hypothesis testing of a specific association. Therefore, effect size and statistical power were not used in the sample size calculation.

### 2.5. Statistical Analysis

Descriptive statistics were used to summarize participant characteristics and study variables. Categorical variables are presented as frequencies and percentages. Normality was assessed using the Kolmogorov–Smirnov test. The data showed significant deviation from normality (*p* < 0.05); therefore, non-parametric tests were used. Scale scores, including Likert-scale items, were treated as continuous variables. Because the data were not normally distributed, medians and interquartile ranges were used as the primary descriptive statistics, while means and standard deviations were retained as supplementary descriptive measures. Group differences in continuous outcomes were assessed using Mann–Whitney U tests or Kruskal–Wallis tests. Associations between categorical variables were assessed using chi-square tests. Spearman correlation coefficients were used to assess associations between continuous scale scores. Internal consistency reliability for PHM-Q, HLS-Q12, and the patient activation items was assessed using Cronbach’s alpha. Because the outcome of participation in preventive health programs was infrequent and the multivariable models showed concerns regarding stability and fit, regression analyses were not retained in the final analysis plan. Accordingly, the final analytical framework was descriptive and unadjusted, and the reported associations should be interpreted as exploratory and hypothesis-generating rather than as independent effects. Statistical significance was defined as *p* < 0.05. Analyses were performed using IBM SPSS Statistics, Version 21.0 (IBM Corp., Armonk, NY, USA). No imputation was performed. Analyses were based on available complete data for the variables included in each analysis.

## 3. Results

### 3.1. Participant Sociodemographic Characteristics (Table 1)

A total of 301 questionnaires were distributed: one participant declined to participate, and 14 questionnaires were excluded due to incomplete responses. These 15 cases were treated as non-responses, giving an overall non-response rate of 4.98% and a final analytic sample of 286 participants. Females constituted most of the sample (65.0%, n = 186). Participants were distributed across age groups, with the largest proportion observed in the 35–54 years age group (46.86%, n = 134). Regarding marital status, over half of participants were married (51.0%), followed by single individuals (31.8%). In terms of educational attainment, most had completed a diploma or bachelor’s degree (42.3%), followed by secondary education (31.8%). Employment status varied, with 41.3% reporting current employment, and 27.3% reporting being unemployed. Most participants resided in Riyadh city (70.3%), while 29.7% lived outside the city. Nearly half of households consisted of 3–5 members (49.3%), and 56.6% of participants reported having at least one chronic disease. Healthcare utilization patterns showed that most reported 0–2 healthcare visits per year (70.6%), whereas 22.4% reported 3–5 visits, and 7.0% reported 6 or more visits annually.

**Table 1:** Participant sociodemographic characteristics.

Variable	Description	N (%)
Gender	Male	100 (35.0)
	Female	186 (65.0)
Age group	18–24 years	57 (19.93)
	25–34 years	52 (18.18)
	35–44 years	67 (23.43)
	45–54 years	67 (23.43)
	55 years or older	43 (15.03)
Social status	Single	91 (31.8)
	Married	146 (51.0)
	Divorced	23 (8.0)
	Widowed	26 (9.1)
Educational level	No formal education	15 (5.2)
	Primary education	12 (4.2)
	Intermediate education	11 (3.8)
	Secondary education	91 (31.8)
	Diploma/bachelor’s degree	121 (42.3)
	Postgraduate studies	36 (12.6)
Occupational status	Student	51 (17.8)
	Unemployed	78 (27.3)
	Employed	118 (41.3)
	Retired	28 (9.8)
	Self-employed	11 (3.8)
Residency	Riyadh city	201 (70.3)
	Outside Riyadh city (Governorate or village)	85 (29.7)
Household size *	1–2	47 (16.4)
	3–5	141 (49.3)
	6 or more	98 (34.3)
Chronic disease	Yes	162 (56.6)
	No	124 (43.4)
Clinic visits per year	0–2 times	202 (70.6)
	3–5 times	64 (22.4)
	6 times or more	20 (7.0)

Analytical sample size (n = 286). * Household size refers to the number of persons living in the household.

### 3.2. HLS-Q12

Mean HLS-Q12 scores were relatively high across items, with an overall mean score of 3.11 (SD 0.46) (Table A2). Mean item scores ranged from 2.81 to 3.36, with most medians equal to 3.00 and narrow interquartile ranges, indicating relatively consistent responses.

### 3.3. PAM-13

Patient activation levels were similarly high across items, with mean scores ranging from 2.94 to 3.45 (Table A3). Median scores were predominantly 3.00, and interquartile ranges were generally narrow. After excluding participants with more than three missing or not applicable responses, the overall mean patient activation item score was 3.22 (SD 0.36). The HLS-Q12 and PAM-13 instruments also showed acceptable reliability (Cronbach’s α = 0.875 and 0.816, respectively).

### 3.4. PHM-Q

Participants generally agreed with the PHM-aligned principles represented in the study-developed questionnaire (Table 2). Item means ranged from 3.68 to 4.08, with medians consistently at 4.00. The overall PHM-Q score was 3.86 (SD 0.57), indicating generally favorable agreement with PHM-aligned principles. The PHM-Q demonstrated good internal consistency (Cronbach’s α = 0.847).

### 3.5. Participation in Preventive Health Programs and PHM Terminology Exposure

Overall participation in preventive health programs was limited (Table 3). Only 17.5% (n = 50) of participants reported having participated in a preventive health program, while most (82.5%, n = 236) reported no prior participation. Familiarity with PHM was limited, with 24.8% (n = 71) of participants reporting prior exposure to the term, compared with 75.2% (n = 215) who had not. Of participants who reported prior exposure to the term PHM, 49.3% (n = 35) reported having a good understanding of the concept.

### 3.6. Correlations Between PHM-Q, HLS-Q12, and PAM-13

Correlation analyses were conducted using participants with complete data on the variables included in each analysis (Table 4). Spearman’s rank correlation coefficients were used. PHM-Q was moderately and positively correlated with HLS-Q12 (ρ = 0.548, *p* < 0.001) and PAM-13 (ρ = 0.433, *p* < 0.001). The strongest association was observed between HLS-Q12 and PAM-13 (ρ = 0.661, *p* < 0.001).

### 3.7. Association Between PHM-Q and Demographic Characteristics

PHM-Q scores differed significantly across several sociodemographic characteristics (Table 5). Participants aged ≥55 years had lower PHM-Q scores than all other age groups. Widowed participants had lower scores than participants in other marital status groups, and married participants scored lower than single participants. PHM-Q scores increased with educational attainment, with postgraduate participants scoring higher than participants in all other education categories, and diploma/bachelor-degree-qualified participants scoring higher than those with primary and lower education levels. Employed participants and students scored higher than unemployed and retired participants. Participants from households with ≥6 members scored lower than those from smaller households. Detailed group distributions and pairwise comparisons are provided in Table A5.

### 3.8. Association Between Participation in Preventive Health Programs and HLS-Q12 or PAM-13

Participants who reported participation in preventive health programs had significantly higher HLS-Q12 scores than those who did not (U = 2745.000, *p* < 0.001) (Table 6). Similarly, PAM-13 scores were significantly higher among participants who reported participating in preventive health programs compared with those who did not (U = 3584.000, *p* = 0.020).

### 3.9. Participation in Preventive Health Programs and Sociodemographic Characteristics

Participation in preventive health programs differed significantly by age, marital status, occupation, residence, and chronic disease status (Table 7). Pairwise comparisons indicated higher participation among younger participants. Specifically, participants aged 18–24 years showed significantly higher participation than those aged 35–44 years (*p* = 0.031), 45–54 years (*p* = 0.001), and ≥55 years (*p* < 0.001). Participants aged 25–34 years also showed significantly higher participation than those aged 45–54 years (*p* = 0.008) and ≥55 years (*p* = 0.003). By marital status, single participants had significantly higher participation than married (*p* = 0.001) and widowed participants (*p* = 0.011). By occupation, participation was significantly higher among students than among those who were unemployed (*p* < 0.001) and among employed participants than among those who were unemployed (*p* = 0.006). In addition, participation was significantly higher among Riyadh city residents than among those living in rural areas (*p* < 0.001) and among participants without chronic disease compared with those with chronic disease (*p* = 0.008). Detailed category levels are provided in Table A4.

## 4. Discussion

This study examined familiarity with the term PHM, agreement with PHM-aligned principles, and participation in preventive health programs among primary care attendees in Riyadh, Saudi Arabia. Familiarity with PHM terminology was limited, whereas agreement with PHM-aligned principles was generally favorable. Higher health literacy and higher patient activation were positively associated with higher PHM-Q scores, and participation in preventive health programs was relatively limited.

Across this sample, familiarity with the term PHM appeared limited, as most participants had not previously encountered the term and only a minority of those exposed reported a good understanding of it. Nevertheless, participants generally expressed favorable agreement with PHM-aligned principles represented in the questionnaire, suggesting favorable attitudes toward PHM-aligned principles despite limited prior familiarity with the specific PHM label. Agreement with PHM-aligned principles varied across several participant characteristics, indicating that agreement with these principles varied across demographic, socioeconomic, and capability-related factors in this sample. These descriptive findings highlight the importance of distinguishing between familiarity with PHM terminology and favorable attitudes toward PHM-aligned principles. This pattern is consistent with a review of PHM definitions and terminology, which indicated that PHM-related terms are often used interchangeably with related population health terms, creating conceptual ambiguity, and that these terms and definitions continue to evolve [23].

It is important to acknowledge a limitation in the construct validity of the PHM-Q. Although the instrument was developed to reflect principles commonly associated with PHM, its items largely represent broad and desirable healthcare principles, including prevention, person-centered care, care coordination, consideration of social determinants of health, value-oriented care, and data-informed improvement. These principles are aligned with but are not specific to PHM as a technical–organizational or operational model. Accordingly, the PHM-Q should be interpreted as reflecting agreement with PHM-aligned principles rather than engagement with PHM-specific activities such as population segmentation, risk stratification, proactive outreach, or formal implementation workflows. High levels of agreement may also partly reflect social desirability or general agreement with positive healthcare principles. In addition, no exploratory or confirmatory psychometric validation was performed beyond internal consistency assessment, and the generally favorable distribution of responses may indicate limited discriminatory capacity and possible ceiling effects. The present study cannot establish that the PHM-Q captures a distinct PHM construct separate from broader favorable attitudes toward prevention-oriented, person-centered, and coordinated care, nor can it determine whether the observed response pattern reflects a coherent PHM-specific latent structure rather than agreement with broadly desirable healthcare values. Accordingly, the contribution of the present study lies primarily in describing familiarity with PHM terminology and attitudes toward PHM-aligned principles within one clinical population, rather than in establishing a validated PHM-specific measure.

This positioning is consistent with empirical work in integrated care and population health management that distinguishes between implementation frameworks, service redesign strategies, and patient-facing perceptions of broader prevention-oriented and coordinated-care principles. The present findings therefore add a descriptive patient-level perspective to this broader literature but do not constitute an implementation-science evaluation of PHM.

Agreement with PHM-aligned principles differed significantly by age, with participants aged ≥55 years showing lower PHM-Q scores than all younger age groups. A similar age-related pattern has been reported, where older adults were less likely than younger adults to report high levels of knowledge about population health-related uses of patient data. A survey conducted in the UK assessed attitudes toward integrated electronic health records used for health care provision, planning and policy, and health research. Although PHM-aligned principles and integrated EHR uses are not the same construct, both assess public or patient receptiveness to broader system-level, data-enabled approaches to healthcare. Support for integrated EHRs was generally favorable overall, although it varied across social groups, with lower support among older participants, and greater indecision among those with lower educational attainment. This provides a conceptually similar example to the present findings, in which agreement with PHM-aligned principles was generally favorable but differed across participant characteristics [24].

PHM-Q scores differed across several socioeconomic indicators, suggesting that agreement with PHM-aligned principles may vary by socioeconomic context. The higher PHM-Q scores observed among participants with greater educational attainment, particularly those with postgraduate education, suggest that education may be associated with greater agreement with PHM-aligned principles. A similar pattern was observed for occupational status, with employed participants and students demonstrating higher PHM-Q scores than those who were unemployed or retired. In contrast, lower PHM-Q scores were observed among married participants compared with single participants, and among those living in larger households. These findings may be considered in relation to Andersen’s Behavioral Model, which highlights the potential relevance of predisposing and enabling factors, including education, employment, and social circumstances. Higher levels of education and occupational engagement may be associated with greater exposure to health information and with greater agreement with PHM-aligned principles. This is consistent with the previously mentioned survey, which found greater indecision among participants with lower educational attainment than among those with a higher degree [24]. Lower PHM-Q scores among married participants and those living in larger households may reflect unmeasured contextual factors. Possible explanations discussed in the prior literature include competing demands on time and attention, but these factors were not directly assessed in the present study.

Health literacy and patient activation were both positively associated with agreement with PHM-aligned principles, with PHM-Q showing moderate positive correlations with HLS-Q12 and PAM-13. This finding is consistent with health literacy frameworks and the patient activation model, both of which suggest that individuals with greater capacity to understand health information and greater confidence in managing their health are more likely to report agreement with PHM-aligned principles. In this sample, the observed associations were consistent with expected relationships described in the prior literature and should be interpreted as descriptive rather than explanatory.

Across participants, participation in preventive health programs was generally limited. Participation in preventive health programs varied significantly across key individual and contextual factors. Previous research has similarly reported limited utilization of some preventive health services in Saudi Arabia. Alqahtani et al. found that awareness of certain preventive services did not always translate into actual utilization, particularly for cancer screening, suggesting that awareness alone may be insufficient to encourage uptake [25].

Because the study did not directly examine why participation differed between participant groups, explanations concerning age, socioeconomic status, chronic disease status, and residence should be interpreted with caution.

An important contextual consideration is that the preventive health programs available in the study setting included screening, vaccination, and counseling services; however, detailed information about the range, frequency, and accessibility of these programs was not formally assessed in this study. Low participation rates may therefore reflect limited program availability or accessibility and individual-level barriers, such as low health literacy or motivation. Future research should more clearly distinguish between supply-side factors, such as service availability, accessibility, and program delivery, and demand-side factors, such as patient awareness, health literacy, motivation, and willingness to participate, when examining preventive health program participation.

Participation in preventive health programs differed significantly by age, with younger participants, particularly those aged 18–34 years, showing greater participation compared with those aged 45 years and older. This pattern may reflect differences in responsiveness to preventive messaging or exposure to health promotion efforts. This descriptive pattern may warrant further investigation in future studies designed to examine barriers to preventive program participation among older age groups. These findings are consistent with prior work suggesting that age is an important factor in the utilization of preventive services [25].

Socioeconomic factors were also associated with participation in preventive health programs. Higher participation among single participants and among working individuals, particularly students and employed participants, compared with married or widowed participants and unemployed individuals, may reflect unmeasured contextual differences between groups. These may include variations in life circumstances, daily demands, and access to supportive environments that influence participation in prevention-oriented activities. Health status was another significant factor, with participants without chronic disease reporting higher participation than those with chronic disease. Similarly, a cross-sectional survey targeting Saudi students studying in the United States found that being married was negatively associated with having routine checkups. On the other hand, the study found that having chronic conditions was positively associated with reports of routine checkups [26]. This pattern may reflect differences in responsiveness to preventive messaging or exposure to health promotion efforts.

Participation in preventive health programs was also higher among Riyadh city residents than among those living in rural areas. In line with our findings, Alfaqeeh et al. highlighted urban–rural differences in access to and utilization of primary health care services, including preventive and health promotion services, among patients in Riyadh Province [27]. This urban–rural difference may be consistent with disparities in access, service availability, transportation, or exposure to public health initiatives described in the prior literature.

Participants who participated in preventive health programs had significantly higher health literacy and patient activation scores than those who did not. Furthermore, this study found that higher health literacy was associated with a greater likelihood of undergoing health checkups or cancer screening, particularly among non-workers [28]. In addition, a study evaluating attendance in the Diabetes Prevention Program found that participation was associated with increased patient activation [29]. Taken together, these findings are consistent with an association between higher health literacy, higher patient activation, and reported participation in preventive health programs, although the cross-sectional and unadjusted nature of the analysis precludes stronger interpretation.

The observed associations are broadly consistent with theoretically expected relationships in health literacy and patient activation research and should therefore be interpreted as descriptive contextual patterns rather than as novel explanatory findings. This study has several limitations. Its cross-sectional design does not allow for causal inference. In addition, the use of convenience sampling may have introduced selection bias. The study was also conducted in a single center, which may limit the external validity and generalizability of the findings. In addition, the relatively high proportion of participants with secondary and higher education may have influenced responses to the PHM-Q, as participants with higher educational attainment may be more likely to understand or agree with PHM-aligned principles. Moreover, the study-developed PHM-Q assessed agreement with broadly PHM-aligned principles rather than technical knowledge of PHM implementation, and the present data cannot establish that it captures a distinct PHM construct separate from broader favorable healthcare attitudes. The present study should not be interpreted as an implementation-science evaluation of PHM, but rather as a descriptive exploratory assessment of familiarity with PHM terminology and agreement with PHM-aligned principles within one clinical population. In addition, because no exploratory or confirmatory factor analysis was performed, and no external validation procedures were undertaken, the dimensional structure and construct distinctiveness of the PHM-Q remain uncertain. The generally favorable distribution of PHM-Q responses may also reflect limited discriminatory capacity and possible ceiling effects, which further support cautious interpretation. Future studies should subject this questionnaire, or a revised successor instrument, to formal psychometric validation before it is used as a distinct measure of agreement with PHM-aligned principles. Furthermore, data were self-reported and may be subject to recall and social desirability bias. Finally, because the final analytical framework was descriptive and bivariate, the reported associations should not be interpreted as independent effects. In the absence of stable multivariable models, residual confounding remains possible, and the observed relationships should be understood as exploratory and hypothesis-generating only.

## 5. Conclusions

This study found limited familiarity with the term population health management among primary care attendees, despite generally favorable agreement with PHM-aligned principles represented in a study-developed questionnaire. Higher health literacy and patient activation were positively associated with PHM-Q scores, and participation in preventive health programs was limited. Because this study used a cross-sectional design, relied on self-reported data, employed an exploratory questionnaire that was not psychometrically validated beyond internal consistency, and retained only descriptive and bivariate analyses, the findings should be interpreted as a context-specific descriptive contribution reflecting attitudes toward PHM-aligned principles and associated patterns within this sample rather than as evidence of formal PHM literacy, independent effects, or readiness for PHM implementation. Future research should use rigorously validated PHM-specific measures and stronger analytical designs to clarify these relationships more definitively.

## Figures and Tables

**Table 2:** Questionnaire Assessing Population Health Management-Aligned Principles (PHM-Q).

Item	Strongly Disagree	Disagree	Neutral	Agree	Strongly Agree	Mean (SD)	Median	IQR
PHM_1	1 (0.3%)	1 (0.3%)	47 (16.4%)	183 (64.0%)	54 (18.9%)	4.01 (0.632)	4.00	0
PHM_2	1 (0.3%)	2 (0.7%)	59 (20.6%)	134 (46.9%)	90 (31.5%)	4.08 (0.759)	4.00	1
PHM_3		5 (1.7%)	70 (24.5%)	144 (50.3%)	67 (23.4%)	3.95 (0.741)	4.00	1
PHM_4		6 (2.1%)	81 (28.3%)	141 (49.3%)	58 (20.3%)	3.88 (0.746)	4.00	1
PHM_5		14 (4.9%)	104 (36.4%)	107 (37.4%)	61 (21.3%)	3.75 (0.845)	4.00	1
PHM_6		18 (6.3%)	109 (38.1%)	106 (37.1%)	53 (18.5%)	3.68 (0.847)	4.00	1
PHM_7		29 (10.1%)	80 (28.0%)	125 (43.7%)	52 (18.2%)	3.70 (0.883)	4.00	1
Total						3.86 (0.57)		

Analytical sample size (n = 286).

**Table 3:** Participation in preventive health programs and PHM terminology exposure.

Item	Yes	No	Not Sure
Have you participated in any preventive health program provided by the clinics (e.g., screening, vaccination, counseling)?	50 (17.5%)	236 (82.5%)	
Have you heard of the term ‘Population Health Management’ before?	71 (24.8%)	215 (75.2%)	
Do you have a good understanding of what Population Health Management means?	35 (49.3%)	11 (15.5%)	25 (35.2%)

Analytical sample size (n = 286).

**Table 4:** Correlations Between PHM-Q, HLS-Q12, and PAM-13.

Variables	Spearman’s ρ	*p*-Value
PHM-Q and PAM-13	0.433	<0.001
PHM-Q and HLS-Q12	0.548	<0.001
PAM-13 and HLS-Q12	0.661	<0.001

Analytical sample size (n = 258).

**Table 5:** Association between PHM-Q and demographic characteristics.

Variable	Test Statistic (df)	*p*-Value
Age group	41.10 (4)	<0.001
Gender	8212.5	0.101
Marital status	32.55 (3)	<0.001
Educational level	49.79 (5)	<0.001
Occupational status	42.48 (4)	<0.001
Residence	4452.0	<0.001
Household size	9.03 (2)	0.011
Chronic disease status	7339.5	<0.001
Healthcare visits/year	1.02 (2)	0.600

Notes: Kruskal–Wallis H test was used for variables with ≥3 categories (reported with df). Mann–Whitney U test was used for two-category variables (reported as U). Analytical sample size (n = 286).

**Table 6:** Association between participation in preventive health programs and HLS-Q12 or PAM-13.

Variable	Preventive Health Program Participation	N	Mean Rank	Mann–Whitney U	*p*-Value
HLS-Q12	Yes	43	173.16	2745.000	<0.001
	No	215	120.77		
PAM-13	Yes	43	153.65	3584.000	0.020
	No	215	124.67		

Analytical sample size (n = 258).

**Table 7:** Association between participation in preventive health programs and sociodemographic characteristics.

Variable	χ^2^ (df)	*p*-Value	Cramer’s V
Age group	23.03 (4)	<0.001	0.284
Gender	1.29 (1)	0.256	0.067
Marital status	14.09 (3)	0.003	0.222
Educational level	7.91 (5)	0.161	0.166
Occupational status	23.81 (4)	<0.001	0.289
Residence	13.69 (1)	<0.001	0.219
Household size	2.88 (2)	0.236	0.100
Chronic disease status	12.65 (1)	<0.001	0.210
Healthcare visits/year	1.32 (2)	0.517	0.068

Analytical sample size (n = 286).

## Data Availability

The data supporting the findings of this study are available from the corresponding author upon reasonable request.

## Abbreviations

The following abbreviations are used in this manuscript:

PHM	Population Health Management
PHM-Q	Questionnaire Assessing Population Health Management-Aligned Principles
HLS-Q12	Health Literacy Survey Questionnaire
PAM-13	Patient Activation Measure
WHO	World Health Organization
NCDs	Noncommunicable diseases
GDP	Gross Domestic Product

## Appendix A

**Table A1:** Questionnaire Assessing Population Health Management-Aligned Principles (PHM-Q) Items.

Item	Statement
PHM_1	Population Health Management focuses on preventing illness rather than only treating it.
PHM_2	Population Health Management aims to improve the health of entire communities, not just individual patients.
PHM_3	In Population Health Management, healthcare providers encourage healthy lifestyle behaviors such as physical activity and following a balanced diet to prevent diseases.
PHM_4	Population Health Management emphasizes coordination and communication among different healthcare providers.
PHM_5	Population Health Management considers people’s living conditions, housing, and income when planning care.
PHM_6	Population Health Management seeks to provide effective, high-quality care while avoiding unnecessary costs.
PHM_7	Population Health Management uses patient and community data to improve healthcare services.

**Table A2:** Health Literacy Survey Questionnaire (HLS-Q12).

Item	Strongly Disagree	Disagree	Agree	Strongly Agree	Mean (SD)	Median	IQR
HL_1	8 (2.8%)	36 (12.6%)	165 (57.7%)	77 (26.9%)	3.08 (0.707)	3.00	1
HL_2	25 (8.7%)	70 (24.5%)	109 (38.1%)	82 (28.7%)	2.87 (0.927)	3.00	2
HL_3	2 (0.7%)	36 (12.6%)	174 (60.8%)	71 (24.8%)	3.11 (0.629)	3.00	1
HL_4	1 (0.3%)	9 (3.1%)	163 (57.0%)	113 (39.5%)	3.36 (0.563)	3.00	1
HL_5	10 (3.5%)	74 (25.9%)	129 (45.1%)	73 (25.5%)	2.93 (0.809)	3.00	2
HL_6	3 (1.0%)	21 (7.3%)	180 (62.9%)	82 (28.7%)	3.19 (0.608)	3.00	1
HL_7	19 (6.6%)	80 (28.0%)	123 (43.0%)	64 (22.4%)	2.81 (0.861)	3.00	1
HL_8	10 (3.5%)	54 (18.9%)	150 (52.4%)	72 (25.2%)	2.99 (0.767)	3.00	1
HL_9	2 (0.7%)	26 (9.1%)	153 (53.5%)	105 (36.7%)	3.26 (0.649)	3.00	1
HL_10	0 (0%)	22 (7.7%)	173 (60.5%)	91 (31.8%)	3.24 (0.582)	3.00	1
HL_11	1 (0.3%)	27 (9.4%)	162 (56.6%)	96 (33.6%)	3.23 (0.626)	3.00	1
HL_12	1 (0.3%)	32 (11.2%)	140 (49.0%)	113 (39.5%)	3.28 (0.670)	3.00	1
Total					3.11 (0.46)		

Analytical sample size (n = 286).

**Table A3:** Patient Activation Measure (PAM-13).

Item	Strongly Disagree	Disagree	Agree	Strongly Agree	N/A	Mean (SD)	Median	IQR
PAM_1	15 (5.8%)	17 (6.6%)	116 (45.0%)	110 (42.6%)	0 (0.0%)	3.24 (0.817)	3.00	1
PAM_2	1 (0.4%)	14 (5.4%)	115 (44.6%)	128 (49.6%)	0 (0.0%)	3.43 (0.616)	4.00	1
PAM_3	2 (0.8%)	14 (5.4%)	168 (65.1%)	73 (28.3%)	1 (0.4%)	3.21 (0.570)	3.00	1
PAM_4	0 (0.0%)	11 (4.3%)	171 (66.3%)	72 (27.9%)	4 (1.6%)	3.24 (0.520)	3.00	1
PAM_5	9 (3.5%)	44 (17.1%)	130 (50.4%)	75 (29.1%)	0 (0.0%)	3.05 (0.775)	3.00	1
PAM_6	1 (0.4%)	8 (3.1%)	124 (48.1%)	125 (48.4%)	0 (0.0%)	3.45 (0.578)	3.00	1
PAM_7	0 (0.0%)	8 (3.1%)	146 (56.6%)	100 (38.8%)	4 (1.6%)	3.36 (0.543)	3.00	1
PAM_8	1 (0.4%)	6 (2.3%)	154 (59.7%)	89 (34.5%)	8 (3.1%)	3.32 (0.541)	3.00	1
PAM_9	0 (0.0%)	13 (5.0%)	159 (61.6%)	75 (29.1%)	11 (4.3%)	3.25 (0.543)	3.00	1
PAM_10	6 (2.3%)	41 (15.9%)	145 (56.2%)	64 (24.8%)	2 (0.8%)	3.04 (0.710)	3.00	0
PAM_11	1 (0.4%)	9 (3.5%)	173 (67.1%)	74 (28.7%)	1 (0.4%)	3.25 (0.529)	3.00	1
PAM_12	4 (1.6%)	47 (18.2%)	135 (52.3%)	71 (27.5%)	1 (0.4%)	3.06 (0.721)	3.00	1
PAM_13	10 (3.9%)	54 (20.9%)	132 (51.2%)	58 (22.5%)	4 (1.6%)	2.94 (0.773)	3.00	1
Total						3.22 (0.356)		

Analytical sample size (n = 258). N/A: not applicable.

**Table A4:** Participation in preventive health programs and sociodemographic characteristics.

Variable	Pairwise Comparison	*p*-Value
Age group	18–24 vs. 25–34	0.669
Age group	18–24 vs. 35–44	0.031
Age group	18–24 vs. 45–54	0.001
Age group	18–24 vs. 55+	<0.001
Age group	25–34 vs. 35–44	0.160
Age group	25–34 vs. 45–54	0.008
Age group	25–34 vs. 55+	0.003
Age group	35–44 vs. 45–54	0.159
Age group	35–44 vs. 55+	0.082
Age group	45–54 vs. 55+	0.644
Marital status	Single vs. married	0.001
Marital status	Single vs. divorced	0.708
Marital status	Single vs. widowed	0.011
Marital status	Married vs. divorced	0.149
Marital status	Married vs. widowed	0.464
Marital status	Divorced vs. widowed	0.080
Occupation status	Student vs. unemployed	<0.001
Occupation status	Student vs. employed	0.100
Occupation status	Student vs. retired	0.006
Occupation status	Student vs. self-employed	0.165
Occupation status	Unemployed vs. employed	0.006
Occupation status	Unemployed vs. retired	1.000
Occupation status	Unemployed vs. self-employed	0.538
Occupation status	Employed vs. retired	0.072
Occupation status	Employed vs. self-employed	0.688
Occupation status	Retired vs. self-employed	0.534
Residency	Riyadh city vs. outside Riyadh city	<0.001
Chronic disease status	Yes vs. no	0.008

Analytical sample size (n = 286).

**Table A5:** Association between (PHM-Q) and demographic characteristics.

Variable	Comparison	*p*-Value
Age group	≥55 vs. 45–54	0.002
Age group	≥55 vs. 35–44	<0.001
Age group	≥55 vs. 18–24	<0.001
Age group	≥55 vs. 25–34	<0.001
Age group	45–54 vs. 35–44	1.000
Age group	45–54 vs. 18–24	0.457
Age group	45–54 vs. 25–34	0.359
Age group	35–44 vs. 18–24	1.000
Age group	35–44 vs. 25–34	1.000
Age group	18–24 vs. 25–34	1.000
Marital status	Widowed vs. married	0.001
Marital status	Widowed vs. divorced	0.001
Marital status	Widowed vs. single	<0.001
Marital status	Married vs. divorced	1.000
Marital status	Married vs. single	0.008
Marital status	Divorced vs. single	1.000
Educational level	No formal education vs. primary	1.000
Educational level	No formal education vs. intermediate	1.000
Educational level	No formal education vs. secondary	0.032
Educational level	No formal education vs. diploma/bachelor’s	0.001
Educational level	No formal education vs. postgraduate	<0.001
Educational level	Primary vs. intermediate	1.000
Educational level	Primary vs. secondary	0.156
Educational level	Primary vs. diploma/bachelor’s	0.007
Educational level	Primary vs. postgraduate	<0.001
Educational level	Intermediate vs. secondary	1.000
Educational level	Intermediate vs. diploma/bachelor’s	0.641
Educational level	Intermediate vs. postgraduate	0.006
Educational level	Secondary vs. diploma/bachelor’s	0.803
Educational level	Secondary vs. postgraduate	<0.001
Educational level	Diploma/bachelor’s vs. postgraduate	0.034
Occupational status	Self-employed vs. retired	1.000
Occupational status	Self-employed vs. unemployed	1.000
Occupational status	Self-employed vs. employed	0.117
Occupational status	Self-employed vs. student	0.085
Occupational status	Retired vs. unemployed	1.000
Occupational status	Retired vs. employed	0.002
Occupational status	Retired vs. student	0.003
Occupational status	Unemployed vs. employed	<0.001
Occupational status	Unemployed vs. student	<0.001
Occupational status	Employed vs. student	1.000
Household size	≥6 vs. 3–5	0.032
Household size	≥6 vs. 1–2	0.033
Household size	3–5 vs. 1–2	1.000

Analytical sample size (n = 286).

## References

[1] United Nations, Department of Economic and Social Affairs, Population Division (2020). World Population Ageing 2019.

[2] Institute for Health Metrics and Evaluation (2025). Global Burden of Disease 2023: Findings from the GBD 2023 Study.

[3] World Health Organization Global Health Expenditure Database. https://apps.who.int/nha/database.

[4] Alqunaibet A., Herbst C.H., El-Saharty S., Algwizani A. (2021). Noncommunicable Diseases in Saudi Arabia: Toward Effective Interventions for Prevention.

[5] Kindig D.A., Stoddart G. (2003). What Is Population Health?. Am. J. Public Health.

[6] Steenkamer B.M., Drewes H.W., Heijink R., Baan C.A., Struijs J.N. (2017). Defining Population Health Management: A Scoping Review of the Literature. Popul. Health Manag..

[7] Matthews M.R., Stroebel R.J., Wallace M.R., Bryan M.J., Swanson J.A., Allen S.V., Bunkers K.S. (2017). Implementation of a Comprehensive Population Health Management Model. Popul. Health Manag..

[8] Struijs J.N., Drewes H.W., Heijink R., Baan C.A. (2015). How to Evaluate Population Management? Transforming the Care Continuum Alliance Population Health Guide toward a Broadly Applicable Analytical Framework. Health Policy.

[9] Steenkamer B., Drewes H., Putters K., van Oers H., Baan C. (2020). Reorganizing and Integrating Public Health, Health Care, Social Care and Wider Public Services: A Theory-Based Framework for Collaborative Adaptive Health Networks to Achieve the Triple Aim. J. Health Serv. Res. Policy.

[10] McGovern L., Miller G., Hughes-Cromwick P. (2014). The Relative Contribution of Multiple Determinants to Health Outcomes. Health Policy Brief.

[11] de Montigny J.G., Desjardins S., Bouchard L. (2019). The Fundamentals of Cross-Sector Collaboration for Social Change to Promote Population Health. Glob. Health Promot..

[12] World Health Organization Regional Office for Europe (2023). Population Health Management in Primary Health Care: A Proactive Approach to Improve Health and Well-Being. Primary Health Care Policy Paper Series.

[13] Cerezo-Cerezo J., de Manuel-Keenoy E., Alton D., Bruijnzeels M., Jurgutis A., Jakab M. (2025). Unlocking the Power of Population Health Management to Strengthen Primary Health Care. Aten. Primaria.

[14] Sørensen K., Van den Broucke S., Fullam J., Doyle G., Pelikan J., Slonska Z., Brand H., HLS-EU Consortium (2012). Health Literacy and Public Health: A Systematic Review and Integration of Definitions and Models. BMC Public Health.

[15] Hibbard J.H., Mahoney E.R., Stockard J., Tusler M. (2005). Development and Testing of a Short Form of the Patient Activation Measure. Health Serv. Res..

[16] Hibbard J.H., Greene J. (2013). What the Evidence Shows About Patient Activation: Better Health Outcomes and Care Experiences; Fewer Data on Costs. Health Aff..

[17] Silberberg M., Martinez-Bianchi V., Lyn M.J. (2019). What Is Population Health?. Prim. Care Clin. Off. Pract..

[18] Mattke S., Hunter L.E., Magnuson M., Arifkhanova A. (2015). Population Health Management and the Second Golden Age of Arab Medicine: Promoting Health, Localizing Knowledge Industries, and Diversifying Economies in the GCC Countries.

[19] van Ede A.F.T.M., Minderhout R.N., Stein K.V., Bruijnzeels M.A. (2023). How to Successfully Implement Population Health Management: A Scoping Review. BMC Health Serv. Res..

[20] Blandi L., Pegollo L., Gentile L., Odone A. (2023). Population Health Management: Principles, Models and Areas of Application in Public Health. Acta Biomed..

[21] NHS England (2018). Population Health Management Flatpack: A Guide to Starting Population Health Management, Version 1.0.

[22] Finbråten H.S., Wilde-Larsson B., Nordström G., Pettersen K.S., Trollvik A., Guttersrud Ø. (2018). Establishing the HLS-Q12 Short Version of the European Health Literacy Survey Questionnaire: Latent Trait Analyses Applying Rasch Modelling and Confirmatory Factor Analysis. BMC Health Serv. Res..

[23] Swarthout M., Bishop M.A. (2017). Population Health Management: Review of Concepts and Definitions. Am. J. Health Syst. Pharm..

[24] Luchenski S.A., Reed J.E., Marston C., Papoutsi C., Majeed A., Bell D. (2013). Patient and Public Views on Electronic Health Records and Their Uses in the United Kingdom: Cross-Sectional Survey. J. Med. Internet Res..

[25] Alqahtani N.G., Alotaibi M.M., Aldhawyan N.M., Afify A., Kofi M., Alqahtani L.G., Alotaibi T.S. (2023). Prevalence and Determinants of Community Members Utilizing Preventive Services Offered by Family Physicians in Riyadh, Saudi Arabia. J. Fam. Med. Prim. Care.

[26] Alzahrani A.M.A., Felix H.C., Stewart M.K., Selig J.P., Swindle T., Abdeldayem M. (2021). Utilization of Routine Medical Checkup and Factors Influencing Use of Routine Medical Checkup among Saudi Students Studying in the USA in 2019. Saudi J. Health Syst. Res..

[27] Alfaqeeh G., Cook E.J., Randhawa G., Ali N. (2017). Access and Utilisation of Primary Health Care Services Comparing Urban and Rural Areas of Riyadh Providence, Kingdom of Saudi Arabia. BMC Health Serv. Res..

[28] Goto E., Ishikawa H., Okuhara T., Kiuchi T. (2019). Relationship of Health Literacy with Utilization of Health-Care Services in a General Japanese Population. Prev. Med. Rep..

[29] Skrine Jeffers K., Castellon-Lopez Y., Grotts J., Mangione C.M., Moin T., Tseng C.H., Turk N., Frosch D.L., Norris K.C., Duke C.C. (2019). Diabetes Prevention Program Attendance Is Associated with Improved Patient Activation: Results from the Prediabetes Informed Decisions and Education (PRIDE) Study. Prev. Med. Rep..

